# SAFE PASSAGES Training Decreased the Incidence of Perineal Trauma During Childbirth

**DOI:** 10.7759/cureus.81974

**Published:** 2025-04-09

**Authors:** Kathleen R Lundeberg, Bart Staat, Jamie D Crosiar, Jason C Massengill

**Affiliations:** 1 Obstetrics and Gynecology, Wright State University Boonshoft School of Medicine, Dayton, USA; 2 Obstetrics and Gynecology, Tri-State Perinatology, Newburgh, USA; 3 3Air Force Medical Service Maternal-Child Patient Safety Program, Brooke Army Medical Center, San Antonio, USA; 4 Obstetrics and Gynecology, Wright-Patterson Air Force Base, Dayton, USA

**Keywords:** anal incontinence, childbirth complications, oasis, operative vaginal delivery, perineal trauma

## Abstract

Background: Active-duty women of childbearing potential comprise a significant portion of service members in the United States (US) Armed Forces. Obstetrical anal sphincter injuries have been associated with significant morbidity including anal incontinence, rectovaginal fistula, and pain. In the early 2000s, the US Air Force OB/GYN (obstetrics and gynecology) leadership championed the development of a novel education intervention that had the potential to standardize maternal obstetrical care across the DoD.

Methods: This prospective cohort design focused on quality improvement of patient outcomes by implementing a regimented training program for obstetrical providers. Baseline rates of perineal trauma in spontaneous and operative vaginal deliveries for each of the Armed Services were established using delivery data from January to December 2010 (n = 272,161). While all three services were given grant funds and education materials, only the US Air Force (USAF) implemented a formal SAFE PASSAGES algorithm and training program from January to December 2012. All but one USAF hospital received on-site training. Perineal trauma rates were compared to post-implementation rates (January 2013-September 2014, with 451,446 deliveries).

Results: The USAF hospitals showed a reduction in the rate of perineal trauma while the other two (Army, Navy) did not. Overall, there was a reduction in Patient Safety Indicator (PSI) 18 (lacerations after operative delivery, p < 0.01) and a statistically insignificant decrease for PSI 19 (lacerations after spontaneous delivery; 5.8%; p = 0.07) across the services. Again, USAF hospitals showed the greatest improvement (41.8%, p < 0.01). In USAF hospitals that received on-site training, PSI 18 rates decreased from 18.5/1000 to 6.0/1000, a 309% improvement (p = 0.012).

Conclusions: The USAF OB/GYN Leadership developed a training program and strategic intervention that is now well-recognized across the Department of Defense and the United States as the standard of care. Decades later, much evidence now supports the tenets of the SAFE PASSAGES training and its historic beginnings.

## Introduction

In 2011, the US Department of Health and Human Services developed the Partnership for Patients (PfP) program to reduce the incidence of iatrogenic patient harm among a spectrum of hospitalized maternal patients. The Department of Defense (DoD) participated in the PfP program initiated by the US Centers for Medicare and Medicaid Services (CMS) [1]. At the time, rates of 3rd and 4th-degree perineal lacerations during childbirth were being studied. The risk of these obstetric anal sphincter injuries (OASIs) was primarily attributed to operative vaginal deliveries and episiotomies [2,3]. A movement in academia navigated towards interventions to reduce perineal lacerations, including now well-known options such as perineal massage, hands-off techniques, warm compresses, maternal positioning, and if episiotomy is medically indicated, doing so in a mediolateral fashion [4-8]. Also, during this time, efforts were directed at questioning the role of routine episiotomy, which is now recognized as lacking in evidence and associated with patient outcomes, preferring a primary cesarean section [9].

The Military Service Surgeon General’s Obstetrics and Gynecology Consultants from the Air Force, Army, and Navy, as well as the Tri-Service Perinatal Advisory Group, recognized this opportunity to reduce iatrogenic harm for active-duty service women, thus supporting mission-readiness. The Air Force leadership developed an algorithm summarizing several evidence-based methods to lower rates of OASIs [10,11]. They implemented a formalized perinatal care team training on the incidence of perineal trauma, cesarean delivery, and neonatal trauma rates in the DoD as a quality improvement strategy to reduce perineal trauma. This quality improvement strategy evolved into leading a standardized change in DoD-wide standard of care.

The insight and initiative in the DoD were groundbreaking for the field of Obstetrics & Gynecology. To date, the data from that project has not been published. We present the data now to demonstrate the research and leadership potential of the DoD in both academia and its ability to change clinical practices.

## Materials and methods

The reviewing Institutional Review Board deemed this Tri-Service quality improvement study exempt. Baseline rates of perineal lacerations for each DoD service branch were established using delivery data reported through the National Perinatal Information Center (NPIC) from January to December 2010 (n = 272,161 deliveries). The standardized patient safety indicators put forward by the US Department of Health and Human Services Agency of Healthcare Research and Quality (AHRQ) were used: Patient Safety Indicator 19 (PSI 19) marked the incidence of third or fourth-degree perineal lacerations occurring during spontaneous vaginal delivery (SVD) and Patient Safety Indicator 18 (PSI 18) identified third and fourth-degree lacerations occurring during operative vaginal delivery (OVD) with vacuum extractors or with obstetric forceps.

**Figure 1:**
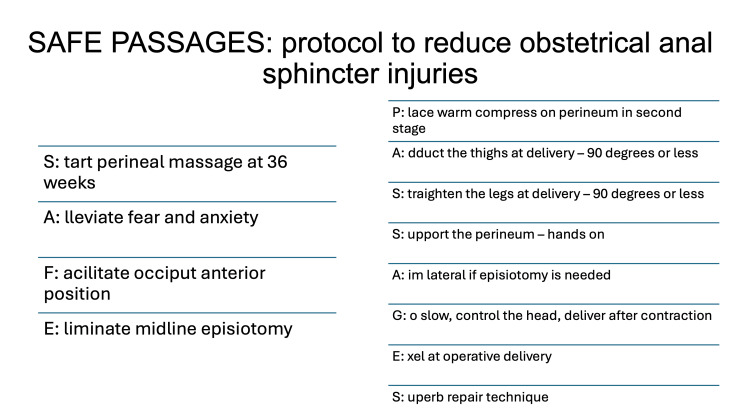
SAFE PASSAGES algorithm This acronym was developed after summarizing evidence-based techniques when educating obstetrical providers with the goal of decreasing perineal trauma.

The Air Force leadership implemented the curriculum which involved on-site or webinar didactic and simulation training in a multi-disciplinary fashion to perinatal care providers involved in vaginal deliveries as well as providing direct instructions on how to appropriately use vacuum and forceps when medically indicated. This training consisted of a four-hour on-site training and then transitioned to a 90-minute interactive webinar for wider accessibility. The session included simulated demonstrations of mediolateral episiotomy, spontaneous vaginal deliveries, and operative deliveries including vacuum and forceps-assisted techniques implementing SAFE PASSAGES principles (Figure 1; for description of the algorithm, see the Appendices). The other two service branches (Army and Navy) monitored and encouraged reduced rates of iatrogenic OASIs but did not provide specific standardized recommendations for reduction.

Implementation of SAFE PASSAGES training took place over 21 months (January 2013 through September 2014). The delivery data was analyzed post-implementation (n = 451,446 deliveries) including only live births and excluding demises. The incidences of PSI 18 and PSI 19 were compared before and during the Partnership for Patients (PrP) intervention year in all three Services. Data was obtained by (i) direct reporting to the Partnership for Patients Implementation Committee of the Air Force, (ii) from hospital discharge coded data at the individual Military Hospital, and (iii) collected in the DoD’s M2 dataset which was summarized by the National Perinatal Information Center (NPIC) contracted to collect and analyze perinatal DoD data. Standard statistical methods were used for comparison, utilizing 2x2 contingency tables for chi-square (with 1 degree of freedom) and t-tests. p-values <0.05 were considered statistically significant.

## Results

Across the three services, there was an overall 19% reduction in PSI 18 (lacerations after operative delivery, p < 0.01) and a nearly significant 5.8% decrease for PSI 19 (lacerations after spontaneous delivery, p=0.066). The only data that remains publicly available is that of the USAF treatment facilities included in this study and is summarized in Table 1. All but one Air Force hospital received SAFE PASSAGES training (17/18, 94%). The USAF hospitals showed the greatest reduction in the rate of OASIs while the other two services did not. In USAF hospitals that received training, PSI 18 rates decreased from 18.5/1000 to 6.0/1000 (X^2^(1, effect size=0.221, N=4776), p = 0.012, Table 1). The single Air Force hospital without onsite or webinar SAFE PASSAGES training had increased rates of PSI 18 and 19. Over the same time, USAF hospitals’ cesarean delivery rates decreased by 1.63% (p <0.05) without a change in neonatal morbidity during the study period.

**Table 1:** Outcomes of participating USAF bases after SAFE PASSAGES training Rates of third- and fourth-degree lacerations compared pre- and post-training, with a decrease in rates of third-degree lacerations with operative vaginal delivery as well as overall PSI 18 rates. SVD: Spontaneous vaginal delivery; OVD: operative vaginal delivery; PSI 19: Patient Safety Indicator 19 incidence of third or fourth-degree perineal lacerations during SVD; PSI 18 incidence of third and fourth-degree lacerations during OVD. *denotes p-value <0.05

	Pre-intervention N=2433	Post-intervention N=2343	p-value
SVD, N (%)	1651 (67.9%)	1580 (67.4%)	0.966
Third degree	18 (1.09%)	23 (1.5%)	0.799
Fourth degree	1 (0.061%)	1 (0.063%)	0.995
PSI 19	19 (1.15%)	24 (1.52%)	0.821
OVD, N (%)	108 (4.44%)	100 (4.27%)	0.954
Third degree	17 (15.7%)	5 (5%)	0.017*
Fourth degree	3 (2.78%)	1 (1%)	0.36
PSI 18	20 (18.5%)	6 (6.0%)	0.012*

## Discussion

OASIs are associated with significant comorbidities including future anal incontinence, rectovaginal fistula, and pain. With the risk of OASI recurrence at subsequent vaginal delivery reported to be 4-8%, weighing the cost of maternal risk, it would require 2.3 cesarean sections to prevent one case of anal incontinence in a woman with prior obstetrical anal sphincter injury [12]. In 2016, most active-duty female service members were of childbearing potential, with an overall live birth rate of 64.9 per 1,000 person-years. Of these deliveries, 75.3% occurred vaginally with perineal lacerations affecting 34.2% not including third- or fourth-degree lacerations [13]. Friedman et al. reported 3.3% and 1.1% of vaginal deliveries in their cohort with third- and fourth-degree lacerations respectively and identified shoulder dystocia and operative vaginal deliveries with or without episiotomy as significant risk factors [2]. To decrease this significant source of maternal morbidity and to address the impact on mission readiness, the US Air Force obstetrical leadership team led the development of a training intervention that held the potential to standardize maternal obstetrical care across the DoD and the United States. It should be noted that at the time of this quality improvement study, evidence of episiotomies, operative vaginal deliveries, and cesarean section morbidity were poorly understood.

The SAFE PASSAGES curriculum was designed for implementation across the Tri-Services involved in obstetrical deliveries and demonstrated efficacy when compared to the service branches without official training. The acronym was developed to remind obstetrical providers of key evidence-based elements to reduce perineal trauma and to minimize midline episiotomies (Figure 1). Developers recognized that vacuum extraction was associated with fewer third-degree lacerations when compared with forceps deliveries, though, with proper training, OASIs could be reduced with forceps as well [3]. At the time, sufficient evidence had been lacking in the utilization of routine episiotomy, but midline episiotomy resulted in more deep perineal tears compared with mediolateral (14.8% versus 7%) with outcomes considered worse than patients with a Pfannenstiel incision [7-9].

The risk of comorbidities following OASIs including anal incontinence, rectovaginal fistulas, and pain led researchers to investigate risk factors and optimize repair. In the early 2000s, the true incidence of OASIs was difficult to estimate due to the lack of uniformity in classification but has been reported to range from 0.1% to 10.9% [14,15]. The American College of Obstetricians and Gynecologists (ACOG) created the reVITALize Obstetric Data Definitions Conference in 2012 to develop and standardize obstetrical clinical data definitions due to the lack of uniformity in classifications of perineal lacerations [15].

During this same period, historical rates of cesarean sections were lower due to the higher utilization of operative delivery and episiotomies. In 2006, ACOG released its first statement on episiotomies at which time 33% of vaginal deliveries were associated with an episiotomy [16]. At that time, the indication for performing episiotomy was anecdotal and deferred to clinical judgment in situations where an expedited second stage of labor was warranted, or the risk of perineal laceration seemed high [16]. This statement was later redacted in 2015 and replaced by the official statement endorsing restrictive episiotomy over universal episiotomy being practiced [17].

The SAFE PASSAGES curriculum was a landmark quality improvement study within the DoD and occurred years before this redacted ACOG statement on episiotomy use. It was part of a larger initiative created by the PfP program and set the stage for future research, and led the change in the standard practice for delivering women. Most recently, a retrospective case-control study utilizing civilian databases analyzed 15,413,957 vaginal deliveries from 2016 through 2021 and showed a total OASIs incidence of 1.1% [18]. In that study, while risk factors remained the same as our cohort study (birth history, operative delivery, and infant weight), it did reflect a substantial decrease in the incidence of previously reported rates of OASIs of 3.3% of third-degree and 1.1% of fourth-degree lacerations [2]. This suggests an improvement in educational training and optimization of obstetrical practices, demonstrating the role of training algorithms such as SAFE PASSAGES.

There are several weaknesses to this study, including a change in the training protocol to universal virtual training sessions during the study period, with one hospital not receiving any training at all. Also, limited data availability for reporting from the other two military branches is an additional weakness. While additional limitations of this study include the delay in publication of findings, a strength of this study includes the demonstration of proof of concept. The SAFE PASSAGES training was replicated at a large civilian multi-hospital system in Northern Virginia with a quasi-experimental pre-post single group design to study the performance of labor and delivery personnel after participating in the program [19]. During those 18 months, 675 personnel participated in the program. Significant improvement was noted in pre-post scores of understandings (59.86%, 93.87%, p<0.0001), performance (36.54%, 93.54%, p<0.0001), and safety culture (3.24, 1.45, 1=high 5=low, p<0.0001). Follow-up provider surveys indicated that 95% had adapted new practice patterns based on the strategies, and severe perineal laceration rates decreased by 38.53% after initiation.

## Conclusions

Significant improvement in the incidence of obstetric perineal trauma occurred without discouraging the use of obstetric vacuum or forceps procedures with standardized perineal trauma reduction training. Implementation of the SAFE PASSAGES program remains a useful tool after implementation in the DoD. The broader implementation using web-accessible formats increased access to standardized training. The implementation of SAFE PASSAGES training in military hospitals reduced the risk of perineal trauma during childbirth without increasing cesarean rates or neonatal harm and led the way in changing standard practice across the United States.

## Appendices

SAFE PASSAGES algorithm

The SAFE PASSAGES algorithm is a clinical training tool integrating evidence-based strategies, personalized interventions to optimize maternal positioning, delivery techniques, and perineal support. This algorithm reminds obstetrical providers of core principles in vaginal and operative vaginal deliveries with the goal of improving maternal outcomes and reduction in OASIs.
